# Are We Able to Prevent Neonatal Readmission? A Retrospective Analysis from a Pediatrics Department in Ploiești, Romania

**DOI:** 10.3390/medicina60050705

**Published:** 2024-04-25

**Authors:** Ioana Roșca, Andreea Teodora Constantin, Daniela Eugenia Popescu, Ana Maria Cristina Jura, Anca Miu, Alina Turenschi

**Affiliations:** 1 Faculty of Midwifery and Nursery, University of Medicine and Pharmacy “Carol Davila”, 020021 Bucharest, Romania; ioana.rosca@umfcd.ro (I.R.);; 2 Neonatology Department, Clinical Hospital of Obstetrics and Gynecology “Prof. Dr. P. Sârbu”, 060251 Bucharest, Romania; 3 Pediatrics Department, National Institute for Mother and Child Health “Alessandrescu-Rusescu”, 010024 Bucharest, Romania; 4 Department of Obstetrics-Gynecology and Neonatology, “Victor Babeș” University of Medicine and Pharmacy, 300041 Timișoara, Romania; 5 Department of Neonatology, Premiere Hospital, Regina Maria Health Network, 300643 Timișoara, Romania; 6 Pediatrics Department, Pediatric Hospital Ploiești, 100097 Ploiești, Romania

**Keywords:** neonatal, readmission, newborns, pediatrics, malnutrition, breastfeeding, multidisciplinary team, public health, inclusion, equity

## Abstract

*Background and Objectives*: Early discharge after childbirth has led to a rise in neonatal readmission, thereby becoming a major concern in recent decades. Our research aimed to identify the risk factors and incidence of neonatal readmission and explore preventive measures. *Materials and Methods*: Our study at the Clinical Hospital of Pediatrics in Ploiești, Romania, included 108 neonates admitted during the neonatal period. *Results*: This accounted for 2.06% of all admissions (5226). The most prevalent cases were malnutrition (25%), fever (20.3%), and bronchiolitis (17.5%). Diarrhea and infectious gastroenteritis were also observed (14.8%), along with acute rhinoconjunctivitis (9.2%) and late-onset sepsis (3.7%). No deaths were recorded. The most significant characteristics identified were number of children (*p* < 0.001) and age at maternity discharge (*p* < 0.001). By following the prevention rules, malnutrition, feeding errors, and infections can be avoided. This includes practicing proper hand hygiene for both mothers and medical staff, as well as educating and demonstrating to mothers the benefits of breastfeeding. In addition, all newborns discharged from the maternity ward would benefit from follow-up at 7–10 days of life. *Conclusions*: Our results confirm the effectiveness of a multidisciplinary team and endorse the promotion of breastfeeding. Implementing quality control measures and regularly evaluating the surveillance program will help improve its effectiveness.

## 1. Introduction

Rehospitalization of neonates within the first month after initial release is a frequent occurrence that causes disturbances for patients and their families, as well as financial strain on healthcare systems. Research on neonatal readmission in the past has concentrated on maternal and neonatal factors [1].

Potentially preventable problems including jaundice and feeding problems account for the majority of hospital readmissions among neonates within 28 days after discharge [2].

In addition to being linked to high rates of morbidity and mortality, the neonatal period is the most crucial time in life for laying a solid foundation for overall health. Because rates of neonatal readmissions can reach 10.1% outside of the US, this is a global concern. Neonatal readmissions incur significant costs for patients, their families, and the healthcare system as a whole. A number of tests and examinations are conducted to determine whether a newborn is ready for discharge from maternity units. Nevertheless, some neonates may experience readmission at any age. For this reason, determining the related maternal and neonatal risk factors is crucial when analyzing how age affects neonatal outcomes, caregivers, and financial obligations [3].

Generally, in Ploiești Maternity Hospital, newborns are discharged by 72 h of life if born via C-section or 48 h of life if born vaginally. If the mother–neonate dyad fits the criteria for discharge, discharge can be earlier than 72 h (i.e., the newborn is breastfeeding, bloodwork and clinical state are normal, and the mother has been informed and trained in the care and resolution of any problems that may arise at home).

## 2. Materials and Methods

We conducted a longitudinal retrospective observational study over a period of one year (November 2022–November 2023) that aimed to evaluate the prevalence of neonates admitted to a pediatric center in Ploiești, Romania, after being discharged from the maternity unit.

The main objectives included the identification and description of the most important diagnoses of hospitalized neonates in the pediatrics unit during the first 28 days of life (the neonatal period corresponds to 0–28 days). The inclusion criteria were: neonates with any signs of disease or poor condition; hospitalized in the pediatric department; and aged up to 28 days, based on clinical or paraclinical data. Patients with late-onset symptoms such as feeding difficulties, fatigue during meals, and growth failure were also included in this study. Exclusion criteria were: incomplete data, and infants who were older than 28 days. Data were collected from the hospital’s electronic register using Microsoft Excel software, version 16.7, U.S. and simple random sampling to reduce bias. The IBM SPSS version 26 software, U.S. was used for data analysis and graphical representation. Normal weight at term refers to a weight of 2800 g to 3999 g at a gestational age between 37 and 42 weeks and prematurity refers to a gestational age below 37 weeks, with subcategories of late preterm (32–37 weeks of gestation), very preterm (28–32 weeks), and extremely preterm (<28 weeks).

A backward regression model was used to estimate relationships between a dependent variable (age at admission) and more independent variables (gender, APGAR score, type of birth, term or preterm neonate, maternal age, number of children, rural/urban residence, age at maternity discharge, and breastfeeding/bottle feeding). This analysis involves starting with all potential predictors and systematically removing those that contribute the least to the model’s predictive power. This approach ensures a more efficient and accurate model by retaining only the most significant variables.

## 3. Results

Our study included 108 neonates admitted to the Pediatric Hospital in the city of Ploiești, who were discharged in good general condition from the maternity ward but presented to the pediatric ward in the immediately following period, up to 28 days of life, with various symptoms specific to the neonatal period but also to the young child.

A quarter of the neonates included in the study (25%) were diagnosed with mild protein-energy malnutrition (PEM), being exclusively breastfed or receiving formula plus the mother’s own milk. Twenty-two cases (20.3%) had fever as the main sign of either infection or dehydration. Nineteen infants (17.5%) had signs and symptoms on clinical examination that were suggestive of bronchiolitis, such as cyanosis, oxygen saturation below 95% (SaO_2_ < 95%), respiratory distress, and wheezing. Due to immaturity and fetal distress, premature babies and those with intrauterine growth restriction (IUGR) were included in the study because these are vulnerable categories for hospitalization in pediatric units during the first period of life. Table 1 describes the main diagnoses of the included patients and the percentage of each category.

For cases with protein-energy malnutrition, we included those neonates who did not regain the 7–10% of birth weight lost during the first two weeks of life or continued to lose weight instead of thriving. Our cases were obtained from the hospital’s database, and the diagnosis coding system allowed us to use PEM for diagnosing neonates with this pathology. Moreover, CDC growth charts were used at admission. Of the 27 neonates discovered with failure to thrive, 10 were exclusively breastfed and 17 were both breast- and formula-fed. Almost a third (33.3%, nine cases) came from lower socioeconomic backgrounds.

A small percentage (five cases, 4.6%) experienced hypernatremic dehydration upon admission owing to the fact that mothers either recognized neonatal warning signs (especially feeding disorders, along with other symptoms described above) or they were advised by family members to rush to the hospital or seek medical aid.

Of the 108 patients admitted to the pediatric unit, all patients presenting with fever or suspected sepsis required blood culture retrieval (*n* = 22). Except for those cases, all patients admitted had peripheral cultures taken, which is the hospital protocol for this category of patients, to ensure early detection of any potential infections. The hospital follows strict procedures to maintain high standards of care and patient safety. Regular monitoring and testing are crucial in identifying and treating conditions promptly. By adhering to these protocols, the medical staff can provide the best possible care for all patients in need.

The late-onset sepsis cases had three positive urine cultures and no negative blood cultures. Generally, upon admission, the first cultures drawn are peripheral, such as nasal and pharyngeal swabs, and central cultures such as blood and urine. Cerebrospinal fluid retrieval was deferred in these cases owing to the fact that the patients responded well to therapy and other cultures were positive, so meningitis was ruled out.

**Figure 1:**
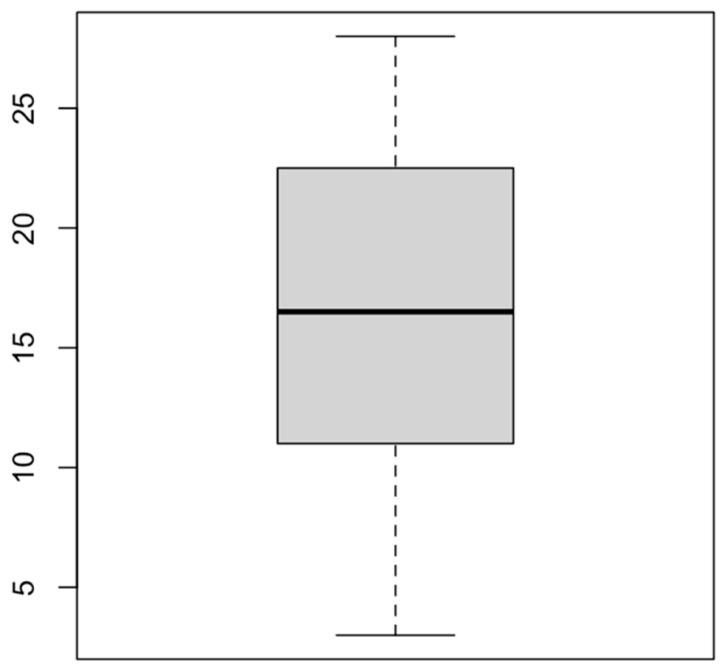
Center and data spread for one variable (readmission age—days).

In this paper, the rate of readmission to the pediatric unit over a period of 1 year (2022–2023) was 5% (108 readmissions), relative to 2140 births that took place in the corresponding year in Ploiești. The readmission age (quantified in days) was observed to be between 3 and 28 days of life, with a mean of 16.57 days and a mode of 13 days. Figure 1 shows the data spread according to the age of readmission for our studied group.

The key aspect of our study was to identify a correlation between different group characteristics and the readmission age. A number of aspects were evaluated, starting from birth parameters, such as gender, Apgar score, type of birth, gestational age, maternal age, number of children, and ending with feeding type (breast or bottle feeds), area of residence, and age in days at discharge from the maternity ward, and these aspects are detailed in Table 2.

Most of our observed group were male (65 vs. 43), with a dominance of an Apgar score between 8 and 10 (88.8%) and a slight difference between cesarean section and vaginal birth (60 vs. 48). The peak age represented in the maternal group was 18–35 years (88.8%), followed by 15 mothers aged >35 (13.8%) and 12 aged <18 years (11.1%). Almost all readmitted newborns were firstborn (77.7%) and from an urban area (70.3%). During maternity stay, nearly all cases were discharged within 2 or 3 days of life (34.2% and 55.5%, respectively), showing no signs of early postnatal complications.

What stands out is the fact that there was a strong correlation between the age of discharge from the maternity ward and the age of readmission (*p* < 0.001), indicating a pattern of early readmission in the pediatric unit for patients who were released early from the maternity unit (between 48 and 72 h of life). At the same time, the number of children (firstborn versus second-, third-, and fifth-born children) seemed to play an important role in readmission (*p* < 0.001). However, some variables mentioned in Table 3 did not have such an impact: the weakest correlation was observed for Apgar score (*p* = 0.95), followed by gestational age. Surprisingly, the types of neonatal feeding, such as breastfeeding, mixed feeding (breastmilk plus formula), and formula feeding alone did not have a great impact on our observed group (*p* = 0.68).

On admission to the pediatric unit based on symptoms, certain paraclinical investigations were performed, namely, complete blood count, C-reactive protein (CRP), peripheral and central cultures, antigens (influenza, respiratory syncytial virus, SARS-CoV-2, rotavirus), and bilirubin levels. As shown in Table 4, bacterial and viral infections were present. Almost a quarter of cases were diagnosed with anemia (15.7%) and 12.9% with hyperbilirubinemia.

**Figure 2:**
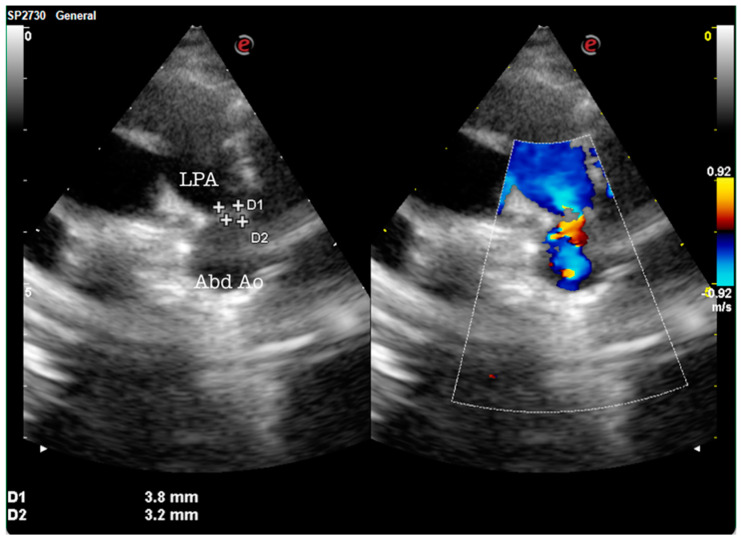
Patent ductus arteriosus (PDA): modified parasternal view showing a moderate tubular/conic patent ductus arteriosus (red flow). LPA—left pulmonary artery; Abd Ao—abdominal aorta.

During the admission and care of the neonates, a multidisciplinary team performed ultrasonographic examinations of all of the readmitted newborns, with careful monitoring of preterm infants and those with IUGR. Of those evaluations, 12 underwent abdominal ultrasound, 19 required cerebral ultrasonography, and 15 required heart ultrasounds. What was interesting was the fact that two of them were diagnosed in the pediatric unit with patent ductus arteriosus, as observed in Figure 2.

The abdominal ultrasonographic examination revealed two cases of grade 1 unilateral hydronephrosis and one case of grade 1 bilateral hydronephrosis, but none of the three cases diagnosed with urinary tract infection (positive urine culture) revealed echographic changes.

As for the cerebral ultrasonographic examination, five neonates were diagnosed with mild hypoxic-ischemic encephalopathy, two with unilateral choroid plexus cysts, and three with intraventricular hemorrhage grade 1.

## 4. Discussion

During the one-year study period (2022–2023), 5226 infants were admitted to our clinic. Of them, 2.1% (108 patients) were neonates who required hospitalization in the pediatric unit after being discharged home from the maternity hospital. The resulting prevalence for newborns was 5%, with 2140 births during the study period. This percentage of newborn babies admitted to a pediatric facility is consistent with worldwide studies reporting newborns as representing between 1 and 2.5% of the total pediatric consultations in the emergency department [4,5,6,7,8]. The moment (age) of neonatal discharge (between 48 and 72 h of life) from the maternity ward was the strongest indicator of early readmission in our study (*p* < 0.001).

Previous research showed that males are more likely to be readmitted compared with females [3]. Although our observations concluded gender does not have such a strong correlation to the age at readmission (*p* = 0.169), our findings are consistent with this affirmation because there is a predominance of male newborns at readmissions (60.1%) and fewer females (39.8%).

First-time mothers face numerous challenges both during their hospital stay and after discharge. Our paper shows an important relationship between firstborns and the age at readmission (*p* < 0.001). The study led by Feenstra M. et al. emphasized the importance of maternal feelings of security and confidence in their maternal role, as they are closely connected to the process of becoming a mother [9]. High-risk pregnancies increase the likelihood of complications and readmission for newborns [10,11,12].

In the present study, although the most prevalent diagnosis was an infection of bacterial or viral origin (bronchiolitis, enterocolitis, sepsis), the primary cause of readmission was represented by growth failure (25%). In a study conducted by Bawazeer M. et al., the most common cause of readmission was respiratory disease, followed by jaundice and fever [3]. Although only one patient was SARS-CoV-2 positive, fever was most common among COVID-19 patients admitted to a pediatric hospital in Bucharest [13]. However, another study reported almost half of their readmissions being due to feeding problems (41%), followed by jaundice [14,15,16].

Only 17.6% of the neonates were successfully breastfed at admission. Most of them were receiving mixed feedings, meaning formula plus breast milk (34.2%), while 33.3% were breastfed but mothers were encountering difficulties. It is concerning that a significant number of neonates did not regain their birth weight loss or continued to lose weight, indicating potential health challenges. The utilization of hospital databases and diagnostic coding systems, along with the use of CDC growth charts, provided a structured approach to identifying and addressing this pathology. The discovery that both exclusively breastfed and mixed-fed neonates were affected underscores the importance of early detection and intervention in cases of failure to thrive. A Romanian study published in 2018 related to feeding practices among Romanian children during the first year of life reported that less than half of the included children were exclusively breastfed for four to six months [17]. Similarly, our group presented with a very low rate of breastfeeding compared with those reported in other countries. In Spain, the breastfeeding rate is as high as 81.7%, whereas it reaches 82% in Germany and 91.1% in Italy [18,19,20]. Our own clinical practice leads us to believe that strong myths concerning breast milk persist among Romania’s population. Mothers who breastfeed are often discouraged by their female family members, usually being told their milk “is not good enough” or it “does not look fatty enough”. Other factors that can contribute to the low rate of breastfed infants could be the short stay in the maternity hospital after birth (48 to 72 h), combined with the high rate of cesarean sections in Romania. In the year 2017, the rate of C-sections in Romania was the second highest in Europe [21]. Although breastfeeding is encouraged in all maternity units, some mothers do not develop appropriate breastfeeding skills during the short hospital stay, and when they arrive home, appropriate support is lacking. Our country does not provide a system for home visits, although family physicians do sometimes offer home visits for newcomers. Lactation consultants are available in our country but only in the private sector and are quite expensive for some families.

There is a pressing need for a more supportive approach to breastfeeding during maternity stays. Many new mothers feel overwhelmed and unsupported during this critical period, which can significantly affect their breastfeeding journey. By creating a supportive environment equipped with knowledgeable staff and resources, we can make a significant difference for these mothers. Providing education, guidance, and emotional support empowers new mothers, boosting their confidence in successfully breastfeeding. Prioritizing this aspect of postpartum care is vital to ensure the well-being of both the mother and newborn. Walsh A. et al. suggested implementing specific steps along with educational programs for both pregnant and postpartum women [22].

Patients with late-onset symptoms such as feeding difficulties and fatigue during meals along with growth failure were included in this study; therefore, a thorough evaluation of newborns who show stagnant growth and feeding difficulties was necessary to exclude cases of late-diagnosed congenital heart disease (CHD) after maternity discharge and to be able to obtain a differential diagnosis [23].

The type of birth did not show a strong correlation with the age at readmission (*p* = 0.65) in our paper, although cesarean birth (CB) has been associated with increased levels of postnatal depression and a negative effect on breastfeeding, suggesting a possible link between maternal depression and neonatal readmission [24].

Despite the fact that some hospitals or physicians recommend neonatal consults after discharge from the maternity ward, it is our strong belief there is a need for a national neonatal follow-up program, which should be implemented in maternity hospitals, especially for high-risk infants. Failure to regain birth weight and thrive can be concerning for neonates. It is important to closely monitor their feeding and growth patterns during the first weeks of life. Those who are struggling to gain weight may need additional support from healthcare professionals to ensure they are receiving adequate nutrition and care, some of them requiring hospitalization in the pediatric unit, which places them at risk of contracting potential infectious diseases or other hospital-acquired infections.

## 5. Conclusions

Our study addressed the main causes and limitations of neonatal readmission and how they can be prevented. Malnutrition, feeding errors, and infections can be avoided by following the prevention rules for nosocomial infections, such as hand hygiene for mothers and medical staff, and by educating mothers on the benefits of breastfeeding. In addition, all newborns discharged from the maternity ward would benefit from follow-up at 7–10 days of life. The examination should be led by a neonatologist for both counseling and supervision of feeding.

Perhaps the most important step in the evolution of newborns admitted to the pediatric unit would be the use of a multidisciplinary team. In our study, newborns received the following consultations: neonatologist, pediatrician, cardiologist, pediatric surgeon, ultrasonography specialist, and pediatric neurologist. This has increased the quality of care, decreased the number of hospital days, and reduced neonatal morbidity and mortality. Our results demonstrate the effectiveness of a multidisciplinary team and support the promotion of breastfeeding, along with neonatal/pediatric surveillance programs as a national public health strategy. Screening for the detection of congenital heart defects (CHD) is crucial in all newborns in the maternity ward using clinical evaluation. If any suspicion of CHD exists, the clinical assessment must be accompanied by paraclinical investigations. Delaying early diagnosis during the neonatal period can lead to an increase in morbidity and mortality associated with this pathology. Therefore, it is essential to implement CHD screening protocols in all maternity hospitals, regardless of their level.

Therefore, it is essential for all doctors, especially neonatologists, to stay up to date with the latest research, guidelines, and techniques related to the diagnosis, treatment, and management of any disease that can impact the newborn after discharge.

Finally, quality control measures should be implemented, and regular evaluation of the surveillance program should be conducted to assess its effectiveness and identify areas for improvement. We hope that our recommendations can serve as a roadmap for policymakers, healthcare professionals, and researchers working to address this critical public health issue.

## Figures and Tables

**Table 1:** Diagnosis of included patients (*n* = 108).

Diagnosis	Number of Patients Diagnosed
Mild protein energy malnutrition	27 (25%)
Fever	22 (20.3%)
Bronchiolitis	19 (17.5%)
Diarrhea and infectious gastroenteritis	16 (14.8%)
Acute rhinoconjunctivitis	10 (9.2%)
Pyodermitis	5 (4.6%)
Late-onset sepsis	4 (3.7%)
Urinary tract infection	3 (2.7%)
Jaundice	1 (0.9%)
Influenza	1 (0.9%)

**Table 2:** Group characteristics.

	Number of Patients (*n* = 108)
Gender	
Male	65 (60.1%)
Female	43 (39.8%)
APGAR score	
8–10	96 (88.8%)
6–7	11 (10.1%)
5	1 (0.92%)
Type of birth	
C-section	60 (55.5%)
Vaginal birth	48 (44.4%)
Maternal age (years)	
<18	12 (11.1%)
18–35	81 (75%)
>35	15 (13.8%)
Number of children	
First child	84 (77.7%)
Second child	21 (19.4%)
Third child	2 (1.85%)
Fifth child	1 (0.92%)
Gestational age and birthweight	
At term with normal birthweight	93 (86.1%)
Preterm	7 (6.48%)
At term with IUGR *	8 (7.4%)
Length of hospital stay (pediatric unit)	
≤5 days	80 (74.1%)
>5 days	28 (25.9%)
Feeding	
Breastfed with no issues	19 (17.6%)
Breastfed with difficulties	36 (33.3%)
Mixed feeding (breastfed and formula)	37 (34.2%)
Formula only	16 (14.8%)
Area	
Urban	76 (70.3%)
Rural	32 (29.6%)
Newborn age at admission	
2–7 days	13 (12.1%)
8–14 days	31 (28.7%)
15–21 days	31 (28.7%)
22–28 days	33 (30.5%)
Newborn age at discharge after birth (Maternity)	
48 h	37 (34.2%)
72 h	60 (55.5%)
4–10 days	9 (8.33%)
>10 days	2 (1.85%)

* IUGR—intrauterine growth restriction.

**Table 3:** The relationship of each characteristic with the age of readmission to the pediatric unit.

Variables	Relationship (*p*-Value) *
Gender	0.169
APGAR score	0.95
Maternal age	0.056
Type of birth	0.65
Number of children	<0.001
Area of residence	0.37
Age (days) at discharge from maternity unit	<0.001
Gestational age	0.76
Type of feeding	0.68

* the alpha value for significance was set at 0.05.

**Table 4:** Investigations and diagnoses of admitted patients.

Investigations and Results	Number of Patients
Umbilical swab	
MRSA *	3 (2.7%)
*Staphylococcus aureus*	1 (0.92%)
*Klebsiella*	2 (1.85%)
Nasal swab	
*Staphylococcus aureus*	20 (18.5%)
Proteus	2 (1.85%)
Group D Streptococcus	1 (0.92%)
Ophthalmic swab—*Staphylococcus aureus*	2 (1.85%)
Urine culture	
*Klebsiella*	3 (2.7%)
*Escherichia coli*	4 (3.7%)
Positive COVID-19 Antigen/PCR **	4 (3.7%)
Positive Type A Influenza Antigen	1 (0.92%)
Positive Respiratory Syncytial Virus (RSV) Antigen	1 (0.92%)
Positive Rotavirus Antigen	2 (1.85%)
High CRP (C-reactive protein) (>5 mg/dL)	16 (14.8%)
Complete blood count revealing anemia (hemoglobin < 12 g/dL, hematocrit < 35%)	17 (15.7%)
Hyperbilirubinemia (total serum bilirubin > 10 mg/dL)	14 (12.9%)

* MRSA—Methicillin-resistant *Staphylococcus aureus*. ** PCR—polymerase chain reaction.

## Data Availability

All data are available in the paper.
